# Methodological Recommendations to Control for Factors Influencing Dream and Nightmare Recall in Clinical and Experimental Studies of Dreaming

**DOI:** 10.3389/fneur.2020.00724

**Published:** 2020-09-15

**Authors:** Benjamin Putois, Wendy Leslie, Claire Asfeld, Caroline Sierro, Susan Higgins, Perrine Ruby

**Affiliations:** ^1^Swiss Distance Learning University, Brig, Switzerland; ^2^Lyon Neuroscience Research Center, CNRS UMR 5292 - INSERM U1028 - Lyon 1 University, Lyon, France; ^3^Clinical Health Psychology, University of Ulster, Ulster, United Kingdom; ^4^Centre Hospitalier Annecy Genevois, Service Pneumologie, Épagny-Metz-Tessy, France

**Keywords:** dream, nightmare, methodology, dream report frequency, experimental control

## Abstract

In order to ensure robust relationships between the dependent and independent variables in clinical dream/nightmare studies, the major factors which influence the frequency of reported dreams must be controlled. This article sets out methodological recommendations to both researchers seeking to ensure the equivalence of experimental groups of participants in group-matching designs, and to clinicians who wish to check that any change in frequency of reported nightmares over the course of a psychological or a pharmacological intervention is not caused by factors other than the experimental treatment itself. The main factors influencing the frequency of dream recall are presented: demographic variables, psychological characteristics, pathological dimensions, and substance consumption. A series of questionnaires is proposed for easily measuring these control variables.

## Introduction

Dreaming is still a mysterious phenomenon and its definition remains controversial and the subject of epistemological debate (1). However, dreams can be studied using a scientific approach (2). The most renowned dream researchers, Hoffman (3) and Schredl (4), emphasized the necessity to increase and improve the use of quantitative methodological practices in dream research to improve results reliability. Quantitative dream studies should systematically control variables which are known to influence some aspects of dreaming, so as to verify that groups of various dreamers are matched in group-matching designs and also to ensure that the results obtained in pre-, post-, and follow-up design studies are due to experimental variables (e.g., patients treated for nightmares vs. patients on the waiting list) and not to confounding variables. For instance, studies testing treatments for nightmares usually compare dream/nightmare report frequency (DRF and NRF) measured at different time points (before and after exposure to the treatment, e.g., imagery rescripting therapy, prazosin) in one group of patients and possibly in one control group (e.g., patients from the waiting-list or exposed to a placebo). For the statistical comparison between groups to be valid, groups must not differ in DRF before the treatment, nor in factors which may influence the evolution of DRF. Consequently, as far as possible, factors influencing DRF should be measured and/or taken into consideration to match the groups to be compared [e.g., (5–12)] and to set the non-inclusion (i.e., psychoactive drugs consumption, and psychopathologies for experimental studies in healthy participants) and exclusion criteria. In pre-post treatment designs, for example, if a patient significantly changed the dose of one of their psychoactive medications (e.g., hypnotics, neuroleptic drugs, antidepressants) during the course of the study, it may have modified DRF & NRF, and this patient's data should be excluded from the analysis [e.g., (13)]. Similarly, if a participant has suffered a trauma during the course of the study, the change in DRF/NRF may be due to the trauma and not to the experimental condition, and their data should therefore be excluded. To improve the quality of future clinical research, outcome studies should also include relevant, and standardized measures known to covariate with some aspects of dreaming.

The objective of this paper is to set out a list of the main variables which should be controlled in dream studies, and more precisely those which influence dream report frequency (DRF), nightmare frequency and emotional valence of dreams. This list includes demographic data, sleep parameters, psychological and cognitive factors, and pathologies and drugs. The authors further recommend validated questionnaires to assess these factors. Note that we do not propose here an exhaustive review and discussion of all the parameters known to covary with some aspects of dreaming. Rather we intend to draw attention on the main variables well-known to influence dream report frequency (DRF), nightmare frequency and the emotional valence of dreams.

## Demographic Variables

### Gender

A meta-analysis comprising more than 40,000 participants showed that women present a slightly higher DRF than men (14). In a retrospective study including several thousand of participants, Nielsen showed that this difference occurs from early adolescence (14 years old) and disappears in middle age (44 years old) (15). This difference is also valid for nightmares except in the older population (older than 60 years old) (16).

### Age

DRF covaries with age (17), it globally decreases with increasing age. An increase in DRF has been described during puberty (10–19 years old) and in early adulthood (19–29 years old) which is followed by a decline. This evolution is modulated by gender. For women, DRF increases in early adolescence, remains at a high level and decreases later in comparison to men (40–49 years old vs. 30–39 for men) (15). A similar profile applies for nightmare frequency, with a peak in childhood followed by a drastic decrease in adulthood. A nightmare rate of at least once a week [a frequency generally thought to reflect moderately severe pathology (18)], is observed in 18% of children and in 9% of adults (19, 20).

*Sex ratio and age are important to match between groups in studies investigating dreams and nightmares*.

## Sleep Variables

Even if counter-intuitive, sleep duration is not a predictive factor of DRF (21). **Sleep stages** are. In average DRF is higher after awakenings in Rapid Eye Movement (REM) sleep (82%) than after awakenings in Non-REM (NREM) sleep (43–67%) (22, 23). Sleep is influenced by **circadian rhythms** which results in a higher DRF at the end of the night than at the beginning of the night irrespective of the sleep stages (24). Dream reports are in average longer, more vivid, story-like and bizarre in the second part of the night. DRF may be influenced by circadian-driven changes in cortical activity (according to increase in body temperature and reduction in melatonin) and by the ultradian rhythm of the NREM/REM cycle (17). **Chronotype** “morningness-eveningness” and nightmare frequency are correlated, especially among women (15).

Intrasleep wakefulness is a decisive parameter regarding DRF (5, 7, 17). The only sleep parameter differentiating high dream recallers (HR) from low dream recallers (LR) is the average **duration of intra-sleep awakenings**. While still in the normal range, HR show longer intra-sleep awakenings (about 2 min on average) than LR (about 0.95 min on average) (7). Long enough awakenings when the dream is still in short term memory appear to be necessary to transfer the short term dream memory into a long term memory (7, 25).

**The time elapsed between awakening and dream report** is also important for the precision and length of the report, the shorter, the better (26).

**The sleeper's environment and sensory stimulation during sleep** may also impact dream activity and notably its emotional content (2). It is for example the case for sounds (27, 28), odors (29, 30), and painful stimuli or thirst (31, 32) also for physical bodily position (33).

*An assessment of wake/sleep rhythms, subjective quantity and quality of sleep using the Pittsburg Sleep Quality Index* (34) *is thus recommended in a dream study. The chronotype can be measured using the short version of the morningness/eveningness questionnaires (MEQ)* (35).

## Psychological Variables

### Influence of Previous Waking Experiences

**Some memories** of wakefulness experiences are incorporated into dreams and are typically incorporated in a partial or indirect way (6, 10). The most recent memories from the day before (day residues) are typically more represented than other recent memories, as are emotional ones, however remote (6, 36, 37). According to some studies, positive and negative emotions occurring during a dream are strongly correlated with the emotional experiences of wakefulness (38). Stress can increase the rate of incorporation of waking memories into dreams (39), and it has also been associated with increased DRF (2, 40, 41) and emotional intensity of dream content (38). For studies investigating emotional dream content, it may thus be necessary to monitor/collect the emotional daily experiences of the participants during the period of dream collection. The Social Readjustment Rating Scale can be used to identify stressful life events (42).

Some researchers suggest that dream recall could be modified by certain conditions and suggestions before falling asleep. However, results are inconsistent. Thoughts or images repressed when falling asleep tend to come back in dreams (43–45). Thought suppression can be measured using the White Bear Suppression Inventory (46).

**Attitudes toward dreams** (ATD), which refers to the general interest in or attention to dreams, is consistently correlated with DRF and the effect of this parameter is large (17, 47). Doing a daily dream diary, which focuses attention on dreaming, is typically associated with a rapid and important increase in DRF. The simple fact of prospectively observing one's own dreams, has also been shown to decrease nightmare frequency (48). The Memory Experiences and Dreams Questionnaire can be used to measure ATD (49).

### Memory

Several types of memory have been tested in HR and LR (short and long term, episodic and semantic, declarative and procedural memory…) but no reliable difference has been identified between the two groups (2). For control purposes, memory capacity can nevertheless be measured using the Mini Mental Score (10 min) (50).

### Personality

**Creativity, openness to experience and alexithymia** are strongly correlated with DRF (2, 40, 51). A high score on an alexithymia scale is associated with difficulty in identifying and describing feelings, limited imaginative processes, and an externally oriented cognitive style (52). The 10-item short version of the Big Five Inventory can be used to measure openness to experience (53).

Subjects with a high DRF are also more likely to have a **personality with thinner boundaries** [Hartmann described people with thin boundaries as being open, trustworthy, vulnerable, and sensitive (52)], to be more **anxious** (54, 55), and to have a higher level of **absorption** [the absorption scale measures the capacity to become absorptively involved in imaginative and esthetic experiences (54, 55)]. The questionnaire assessing thin and thick personality boundaries has been developed by Hartmann (56). Absorption can be assessed with the absorption scale (34 items) (57). **Neuroticism** is correlated with nightmare frequency (58).

## Methods for Studying Dreams

Several methods are classically used to assess some aspect of dreaming (retrospective or prospective DRF questionnaires, dream diaries with written or oral reports, and laboratory awakenings usually followed by an oral report), and these different methods result in different quantitative and qualitative measures (59–61). It is therefore important to use the same dream recording method in participants from different groups supposed to be compared. Retrospective measures are better correlated with prospective measurements using a dream logbook, compared to those using questionnaires for home measurements (62). The correlation between questionnaires and diaries measures are high. Nevertheless, recording a dream diary increases DRF in low and medium dream recallers, and decreases DRF in high dream recallers (63). It would therefore be relevant to measure dream recall frequency at baseline using a retrospective questionnaire.

Recording dreams using the method of provoked awakening in the laboratory–using a voice recording and the sudden awakening method (26)—is the best controlled method. The awakening method itself can influence DRF (abrupt awakenings favor dream recall compared to gradual awakening) (64). Oral reports using a voice recorder device are indeed longer (61). It is probably because oral report are less effortful and can be done right after awakening. When possible to control, the time lapse between awakening and the dream report should indeed be minimized in order to diminish memory loss and reconstruction bias.

## Pathologies and Medications

### Sleep Disorders

According to the existing literature, the main sleep disorders known to be associated with variations in DRF and/or negative dream emotions are insomnia, narcolepsy, nightmare disorders, and somnambulism or night terrors. There is less evidence for such variations in sleep-related breathing disorders (SBD) and restless legs syndrome (65, 66). The variations in dream frequency in patients suffering from idiopathic hypersomnia, REM sleep behavior disorder, and NREM parasomnia are less clear since fewer studies have been done in these pathologies (66, 67).

According to some studies, in primary **insomnia**, deterioration of sleep (decreased total sleep time, REM and NREM sleep duration, and sleep efficiency) is associated with a decreased frequency of dream and nightmare reports (68). However, several other studies have reported an increased DRF in insomnia patients (66, 69). In any case, insomnia is worth evaluating in dream/nightmare studies and its intensity can be measured using the Insomnia Severity Index (70).

**Narcolepsy** severity is correlated with an increase in DRF and higher occurrence of nightmares and lucid dreams (71, 72). Dream content in patients with narcolepsy is more bizarre, with a more negative emotional tone (73). The symptoms suggestive of this rare disease are uncontrollable sleep attacks, cataplexy, sleep paralysis, and hypnagogic or hypnopompic hallucinations.

**Nightmare disorders** are characterized by an increase in both dream and nightmare recall (66). And as DRF and nightmare frequency are usually positively correlated (20) both parameters are worth measuring in dream and nightmare studies.

**In somnambulism and night terrors** no variations in DRF has been observed but an increase in nightmare frequency or negative dream emotions has been reported (66). Night terrors are mainly observed in children, and sign of somnambulism can be provided by partners in adults.

Some studies have reported an increased DRF and duration of dreams in patients with **SBD** (74). Dreams are more negative in sleepers with an apnea-hypopnea index >15 (75). Continuous positive airway pressure therapy (Gold Standard treatment of SBD) decreases nightmare frequency in SBD patients suffering from traumatic disorders (76). In the absence of a polygraph or polysomnography, the Berlin questionnaire or Epworth Somnolence Scale (77) may be used as an indicator of SBD risk.

The severity of **restless legs syndrome** may be correlated with DRF, but a review of the literature shows no effect of this syndrome on dreaming (78). The Restless Legs Syndrome Rating Scale may be used to assess this disorder (79).

*Preliminary screening should be carried out, especially for insomnia, narcolepsy and nightmare disorders*.

### Psychopathologies

Traumas are associated with nightmares and disruptive nocturnal behaviors (80). Nightmares are the most frequent symptom of **post-traumatic stress disorder** (PTSD) (81). Dreams are less frequent but significantly more threatening and hostile (82). Nightmare frequency is correlated with PTSD severity (83). Patients with PTSD experience significantly more replicative (i.e., reproducing the traumatic experience) nightmares (46%) than other patients (11%) (84). PTSD severity can be measured using the Post-traumatic Stress Disorder Checklist Scale (85) or the revised Impact of Event Scale (86).

Sleep related symptoms of **depression** include sleep fragmentation, early morning awakening, decreased rapid eye movement (REM) sleep latency and increased rapid eyes movements density in REM sleep (87). Decrease in DRF and intensity of negative emotions in dreams could be linked to the severity of symptoms (88, 89). Depression severity can be measured using the short form of the Beck Depression Inventory II (90).

Alteration of REM sleep in **anxiety disorders** is still the subject of debate (91). Elderly patients suffering from generalized anxiety disorder have more nightmares (91). The Spielberger State-Trait Anxiety scale measures the state and trait anxiety (92), a short version is also available (93).

**Schizophrenic patients** have stranger dreams, with stronger negative emotional valence compared to healthy subjects. However, DRF is not modified in these patients (94). Note that schizophrenia, as all other neurologic or psychiatric pathologies, is classically an exclusion criterion in dream studies targeting the healthy population.

*In order to decrease heterogeneity among group participants in studies investigating dreams in patients, preliminary screening should be carried out, especially for post-traumatic stress disorders and depression*.

### Substances

Insomniac patients treated with benzodiazepine have lower DRF (69). Flunitrazepam leads to more aggressive, sexual and unpleasant dream content (95). Gradual reduction of doses of clonazepam increases nightmares in some patients (96). Z-drugs (e.g., zolpidem, zopiclone) can also modify both dream content and frequency. More generally, **hypnotic drugs** tend to decrease DRF (for a review on the effect of psychotropic drugs on dreaming, see Nicolas & Ruby in the same issue).

Most **antidepressants** increase sleep continuity and reduce intra-sleep awakenings, which may explain their reductive effect on DRF (see Nicolas and Ruby in the same issue).

**Alcohol** affects sleep quality and dreams. An acute pre-bedtime dose of alcohol in non-alcohol-dependent individuals deteriorates REM sleep (97, 98). There is debate about its effect: several studies have observed longer latency in REM sleep and decreased REM sleep only in the first half of the night, while other studies find this effect across the whole night (99–101). The link between alcohol at bedtime in non-alcoholic people and DRF does not seem to have been studied. Alcohol-dependent subjects report more negative dreams than those who are non-alcohol-dependent (102), with an increase in dream emotional negativity during the first weeks of abstinence (103). It may therefore be important to report and/or control alcohol consumption habits in dreams studies.

**Cannabis** increases the duration and latency of REM sleep (104). Intensification of vivid dreams is a cardinal symptom of cannabis withdrawal (104). Both non-clinical and clinical research has now characterized the profile of cannabis withdrawal, with sleep disturbances and vivid dreams representing its hallmark symptoms.

Given that the use of nicotine patches increases DRF (105), as does varenicline (used to help quit smoking) (106) one may expect that **tobacco** would influence DRF, however, this remains to be tested experimentally.

**Prazosin** is an alpha blocker recommended for the treatment of chronic nightmares in PTSD patients (107–109), and in this case, inhibition of the sympathetic nervous system may desensitize memories of fear which could explain the reduction of nightmare frequency induced by this treatment. To our knowledge, the impact of Prazosin on DRF is unknown. Other substances have been proposed for treating nightmares, but the current level of proof of their effectiveness is too low to be recommended (110).

*Comparing groups in dream studies therefore requires consumption of the following substances to be controlled: benzodiazepine, antidepressant medication, prazosin, alcohol and cannabis. For longitudinal studies, the regularity of intake during the study should be monitored and any significant change in intake should be an exclusion criterion*.

## Conclusion

This article does not aim to review and discuss all the variables which are known to influence dream and nightmare recall frequency. Rather our objective is to provide a list of the main variables that are currently well-known to influence dream and nightmare recall frequency, and which, therefore, would be necessary to monitor or control in clinical studies investigating dreams. The authors therefore aim to set out methodological recommendations for conducting clinical studies on dreams and nightmares. It is essential for studies comparing different groups of dreamers to ensure that observed differences are due to experimental variables and not to uncontrolled differences between dreamers.

This methodological overview is nevertheless limited by the current literature on dream research i.e., the limited existing literature on the influence certain variables have on dreams. The influence of some parameters has been scarcely studied and the influence of some other parameters are debated in the dream research community. Table 1 summarizes the variables known to covary with DRF and dreams emotional valence/intensity and presents the reliability of the effects according to the current knowledge. The main recommendations resulting from this overview of the literature are that (1) in clinical studies, medical interviews should include a differential diagnosis for traumatic disorders, sleep disorders and mood disorders, and these disorders should be assessed in case of group comparison, (2) DRF should be measured in addition to NRF in longitudinal studies on nightmares, as well as psychoactive drug consumption (notably alcohol, antidepressant, cannabis, and prazosin), and significant variations in the consumption of psychoactive drugs during the course of the study should be considered as an exclusion criterion, (3) dreamers' groups to be compared should be matched for the maximum of the parameters covariating with DRF, notably: age, sex ratio, psychopathologies, and psychoactive drug consumption.

**Table 1:** Variables known to covary with dream recall frequency or dreams emotional valence/intensity.

**Variables**	**Available measure to assess the variable**	**Items number**	**Cut-off**	**Reliability of the covariation**
**Demography:**				
Age				^***^
Gender				^***^
**Sleep:**				
Quality	Pittsburgh sleep quality index	17	Yes	^***^
Chronotype	Morningness/eveningness questionnaires	5	Yes	^**^
Insomnia	Insomnia severity index	5	Yes	^**^
Apnea	Berlin questionnaire (apnea risk) or Epworth somnolence scale	11 8	Yes Yes	^*^
**Psychopathology:**				
Trauma	PTSD checklist	17	Yes	^***^
	Impact of event scale	22	Yes	
Depression	Beck depression inventory 2–short version	13	Yes	^***^
Anxiety	State-trait anxiety inventory–short version	6		^*^
**Psychology:**				
Stress	Social readjustment rating scale–short form	47	Yes	^*^
Suppression	White bear suppression inventory	15		^*^
Personality	Big five inventory–short version	10	Yes	^**^
	Hartmann's boundary questionnaire	18		
	Absorption scale, Tellegen absorption subscale and Atkinson's personality inventory	34		
**Substances:**				
Alcohol				^***^
Psychotropic drugs				^***^
Cannabis				^***^
Prazosin				^***^

*Number of items refers to the number of questions in the scale*.

*Cut off refers to a score that is presumed to represent the boundary between “normal” and the “clinical range” on an outcome measure*.

*** *Robust scientific literature, several decades of research, existence of a literary review, existence of several decades of explanatory theories*.

** *Relatively abundant scientific literature, several decades of research, existence of a literary review, lack of explanatory theories*.

* *Very recent studies or existence of inconsistent experimental results*.

With this methodological perspective we aim at increasing awareness of the need to control for several variables in clinical dream and nightmare studies and to stimulate discussion amongst experts in the field. A consortium could further develop this avenue of research to establish comprehensive guidelines and recommendations on the subject. As a first step, we propose here a list of variables currently known to influence DRF, nightmare frequency and the emotional valence of dreams—and which it may be necessary to monitor or control in clinical studies investigating dreams—and means to measure these variables. This article is intended to help improving methodological practices in clinical studies on dreams and nightmares, and is dedicated to clinician non-expert in sleep and dream science. This article may thus help to ensure that in future clinical studies, the observed differences between groups of dreamers are due to the experimental variables manipulated and not to confounding factors.

## Author Contributions

BP developed the research subject presented herein and wrote the manuscript, with the support of CA, CS, SH, and WL under the supervision of PR. PR critically revised the manuscript and added important intellectual content. All authors contributed to the article and approved the submitted version.

## Conflict of Interest

The authors declare that the research was conducted in the absence of any commercial or financial relationships that could be construed as a potential conflict of interest.
